# The effectiveness and safety of corticosteroid therapy for IgA nephropathy with crescents: a prospective, randomized, controlled study

**DOI:** 10.1186/s12882-022-02661-6

**Published:** 2022-01-21

**Authors:** Mengjun Liang, Liping Xiong, Aihua Li, Jiafan Zhou, Yajuan Huang, Miaofang Huang, Xing Zhang, Hongrui Shi, Ning Su, Yi Wei, Zongpei Jiang

**Affiliations:** Department of Nephrology, the Sixth Affiliated Hospital, Sun Yat-sen University, 26 Yuancun Er Heng Road, Guangzhou, 510655 China

**Keywords:** IgA nephropathy, Crescents, Corticosteroid therapy

## Abstract

**Background:**

Pozzi protocol (methylprednisolone intravenous infusion in 1st-3rd-5th months and oral steroid for 6 months) has been thought to be the classic therapy for IgA nephropathy (IgAN) patients with proteinuria> 1.0 g/24 h. There is no consensus on the treatments for IgAN with active pathological changes, especially for IgAN patients with crescents proportion < 50%. This study aimed to evaluate the effectiveness and safety of the treatment protocol of methylprednisolone intravenous infusion at the 1st-2nd-3rd months for IgAN patients with crescents.

**Methods:**

In this prospective, randomized, controlled, non-blind study, 68 IgAN patients with crescents proportion < 50% were divided into the 1–2-3 group receiving 0.25 g/d methylprednisolone intravenously for 3 consecutive days in the 1st-2nd-3rd months, and oral prednisone 0.5 mg/kg/d on consecutive days for 6 months and the 1–3-5 group with the same intravenous methylprednisolone treatment in the 1st-3rd-5th months, and the same oral prednisone. The primary outcome measure was remission of proteinuria (complete or partial); while the secondary outcome measures were deterioration of renal function (evidenced by a 50% rise from baseline serum creatinine levels, or a 25% decline from baseline eGFR levels).

**Results:**

There was no significant difference in incidence of crescents (median 14.66% vs. 11.45%, *p* = 0.143) between the 1–2-3 and 1–3-5 groups. From month 1 to month 6, there was a decreasing trend in the levels of urine protein and serum creatinine and an increasing trend in eGFR in both groups. The mean period of remission in the 1–2-3 group seemed shorter. Overall, there were 55 (80.89%) patients meeting remission. The rates of remission in the 1–2-3 group and 1–3-5 group were 85.3 and 76.47%, respectively (*P* = 0.644). The 1–2-3 group had lower creatinine and higher eGFR than the 1–3-5 group, but the difference was not significant. The complication rate was 11.11 and 15.79% in the two groups, respectively. There was no significant difference in the complications between groups.

**Conclusions:**

Both the 1st-3rd-5th and 1st-2nd-3rd protocols can effectively alleviate proteinuria and protect renal function in IgAN patients with crescents but the 1st-2nd-3rd protocol seemed to have better effectiveness.

**Trial registration:**

ClinicalTrials.gov, Identifier: NCT02160132, Registered June 10, 2014.

## Background

IgA nephropathy (IgAN) is the most common type of primary glomerulonephritis, accounting for about 45% of glomerulonephritis in China [1], and up to 50% of IgAN patients develop end-stage renal disease (ESRD) within 20 years after. Risk factors for the progression of IgAN include onset proteinuria> 1.0 g/24 h, hypertension, renal insufficiency, severe pathological changes (such as glomerular sclerosis, tubule atrophy and interstitial fibrosis), and persistent proteinuria during follow-up are independent predictors of IgAN progression [2, 3]. A recent study has demonstrated that severe renal histological injury may be observed in some IgAN patients with benign clinical characteristics [4].

The new Oxford classification has introduced the crescent score (the MEST-C score) in the IgAN pathological analysis to predict renal prognosis of IgAN, and the study has demonstrated that even the proportion of crescent less than 25% has a significant impact on the prognosis [5, 6]. Current researches showed that among the biopsy-confirmed IgAN patients, 19.6% presented with C1 and 1.8% with C2 scores [7], and crescents in C1 have an increased risk of poor outcome without immunosuppression [8].

The 2012 Kidney Disease Improving Global Outcome (KDIGO) guidelines [9] recommend corticosteroid therapy (Pozzi protocol) for IgAN patients with persistent proteinuria> 1.0 g/24 h and preserved renal function (estimated Glomerular filtration rate [eGFR] > 50 ml/min/1.73m^2^), including intravenous infusion of methylprednisolone for 3 consecutive days in the first, third, and fifth (1st-3rd-5th) months and a half dose of oral prednisone every other day for 6 months. The study with 10-year follow-up showed that this Pozzi protocol is beneficial to reduce proteinuria and protect kidney function [10, 11].

The 2012 KDIGO guidelines recommend that crescentic IgAN (> 50% glomeruli with crescents with rapid deterioration in eGFR) can be treated with corticosteroid and immunosuppressive agents [9]. But there is no guideline regarding corticosteroid therapy for IgAN patients with crescents proportion < 50%, even < 25% (C1) currently. It has been shown that early corticosteroid therapy is beneficial to reverse the active pathological changes in IgAN [12, 13], and reducing the dosage of corticosteroids can reduce the side effects [14].

Considering the timeline of disease progression of active crescents, we hypothesized that more aggressive corticosteroid therapy, such as changing the timing of corticosteroid therapy from the first, third, and fifth (1st-3rd-5th) months to the first, second, and third (1st-2nd-3rd) months, may bring more therapeutic benefits for IgAN patients.

To test this hypothesis, we conducted a prospective, randomized, controlled study to evaluate the effectiveness and safety of the treatment protocol of methylprednisolone intravenous infusion at the 1st-2nd-3rd months for the IgAN patients with crescents by compared with the conventional Pozzi protocol of methylprednisolone intravenous infusion at the 1st-3rd-5th months.

## Methods

### Study design and participants

This was a prospective, randomized, controlled, non-blind study. IgAN patients with crescents were enrolled between January 2017 to December 2019 in our clinic medical center.

The inclusion criteria were: 1) Age 14–65 years, regardless of sex; 2) Clinical evaluation and renal biopsy diagnostic for primary IgAN, presenting with crescents; 3) Mean urinary protein excretion of 0.5–3.5 g/24 h on two successive examinations; 4) eGFR≥50 ml/min/1.73 m^2^; 5) Willingness to sign an informed consent. The exclusion criteria were: 1) Secondary IgAN, such as systemic lupus erythematosus, Henoch-Schonlein purpuric nephritis and hepatitis B-associated nephritis; 2) Proportion of crescent ≥50%; 3) Pure fibrous crescents; 4) biopsies with the number of glomeruli < 8; 5) Current or recent (within 30 days) exposure to high-dose of steroids or immunosuppressive therapy; 6) Date of renal biopsy exceeded more than 30 days; 7) Cirrhosis, chronic active liver disease; 8) History of significant gastrointestinal disorders (e.g. severe chronic diarrhea or active peptic ulcer disease); 9) Any active systemic infection or history of serious infection within 1 month; 10) Other major organ system disease (e.g. serious cardiovascular diseases including congestive heart failure, chronic obstructive pulmonary disease, asthma requiring oral steroid treatment or central nervous system diseases); 11) Active tuberculosis; 12) Malignant hypertension that was difficult to be controlled by oral drugs; 13) Known allergy, contraindication or intolerance to the steroids; 14) Pregnancy or breast feeding at the time of entry or unwillingness to comply with measures for contraception; 15) Malignant tumors; 16) Mental aberrations; 17) Current or recent (within 30 days) exposure to any other investigational drugs.

The statistical software SAS® 9.4 (SAS Institute) was used to generate the assignment list and the random numbers were placed in separate sealed envelopes by the statistical assistant in our medical center. We screened all the patients meeting the eligibility criteria and enrolled the participants. The randomization and allocation procedures were carried out the research assistant based on the random number obtained from the sealed envelopes. All the enrolled participants were randomly assigned in a 1:1 ratio to the 1–2-3 group (received methylprednisolone intravenous infusion at the 1st-2nd-3rd months) or the 1–3-5 group (received methylprednisolone intravenous infusion at the 1st-3rd-5th months). Treatment allocation was not masked.

Patients in the 1–2-3 group received methylprednisolone 0.25 g/d intravenously for 3 consecutive days in the 1st-2nd-3rd month, and oral prednisone 0.5 mg/kg/d on consecutive days for 6 months and those in 1–3-5 group were treated with methylprednisolone 0.25 g/d intravenously for 3 consecutive days in the 1st-3rd-5th month, and oral prednisone 0.5 mg/kg/d on consecutive days for 6 months. During the 6-month therapy period, patients were recommended to receive blockers of the renin-angiotensin system for comprehensive supportive care and to reduce blood pressure to the target of below 125/75 mmHg. No patient in this study received any other immunosuppression, such as mycophenolate, cyclophosphamide, or cyclosporine A.

This study was conducted in accordance with the Declaration of Helsinki and approved by the institutional review board of our hospital (approval no. 2014ZSLYEC-009). Written informed consent was obtained from the patients before enrollment. The trial in the current study was registered to the ClinicalTrials.gov (NCT02160132).

### Clinical and pathological evaluation

Proteinuria was defined as urine protein for 24 h on two successive examinations during every follow-up. Serum creatinine was tested by sarcosine oxidase method. Estimated glomerular filtration rate (eGFR) = 175 × serum creatinine (mg/dl)^-1.234^ × age (year)^-0.179^ [if female,× 0.79], MDRD equation [15]. Renal biopsy was performed in every patient and the biopsy specimens were reviewed by one pathologist who was unaware of clinical details of the patients. All the samples were divided into three parts for light, immunofluorescence and electron microscope examinations. Total number of glomeruli, mesangial proliferation, endocapillary hypercellularity, segmental glomerulosclerosis, interstitial inflammatory infiltration had been recorded for the MEST score. Cellular and/or fibrocellular crescents were recorded and taken to MEST-C score classification; but fibrous crescents were not considered [6]. Lee’s grade was divided into grades II, III, IV, V according to Lee’s glomerular grading system [16].

### Outcome measures

All participants were followed up for 6 months. The primary outcome measure was remission of proteinuria (complete or partial); while the secondary outcome measures were deterioration of renal function (evidenced by a 50% rise from baseline serum creatinine levels, or a 25% decline from baseline eGFR levels, or onset of end-stage renal disease). Treatment outcomes included complete remission (CR), partial remission (PR), and no response (NR). CR was defined as proteinuria< 0.3 g/24 h, without active urinary sediment, serum albumin≥35 g/L, serum creatinine stable within 15% of the basal level. PR was defined as proteinuria decreased by more than 50% of the basal level, serum albumin≥30 g/L, and serum creatinine stable within 15% of the basal level. NR was defined as the decrease of proteinuria not exceeding 50% of the basal level, and the serum albumin< 30 g /L. Steroid therapy-related side effects were recorded, including steroid diabetes, upper respiratory tract infection, pneumonia and skin-related infection. Steroid diabetes was defined as no previous history of diabetes, and an increase in blood sugar level that meets the diagnostic criteria for diabetes during corticosteroid therapy.

### Statistical analysis

Per protocol set (PPS) was used for analysis of effectiveness and safety set (SS) was used for analysis of safety. Continuous data with normal distribution (eg. age, proteinuria and serum creatitine) were indicated with mean ± standard deviation (SD) while categorical data were indicated with number and percentage (%). Student’s independent t-test was used to compare the mean differences between 1 and 2-3 and 1–3-5 groups. Categorical variables were compared using the Chi-square test or Fisher’s exact test (if the expected value ≤5 was found). Data without normal distribution (eg. crescents) were presented as the median (quartile) and were compared with non-parametric tests. Repeated measurement ANOVA was used to investigate the significances of changes in proteinuria, serum creatinine, and eGFR over time. Kaplan-Meier survival function and log-rank test were used to compare the developing trends of total remission (CR + PR) and CR-only between two groups. To further investigate the association between group variable and outcome index, proteinuria, serum creatinine, and eGFR, a linear regression under generalized estimating equation (GEE) model was used. All patient’s results were measured at 6 time-points: month 1, 2, 3, 4, 5, and 6. An autoregressive (lag1) correlation matrix was adopted for the repeated measure data. The linear regression coefficient B was reported.

A *P* < 0.05 would be recognized as reaching the significance of each test, two-tailed. All analyses were performed using IBM SPSS Version 25 (SPSS Statistics V25, IBM Corporation, Somers, New York).

This clinical trial adheres to CONSORT guidelines. A completed CONSORT checklist has been provided as Appendix.

## Results

### Patient’s clinical characteristics

**Fig. 1:**
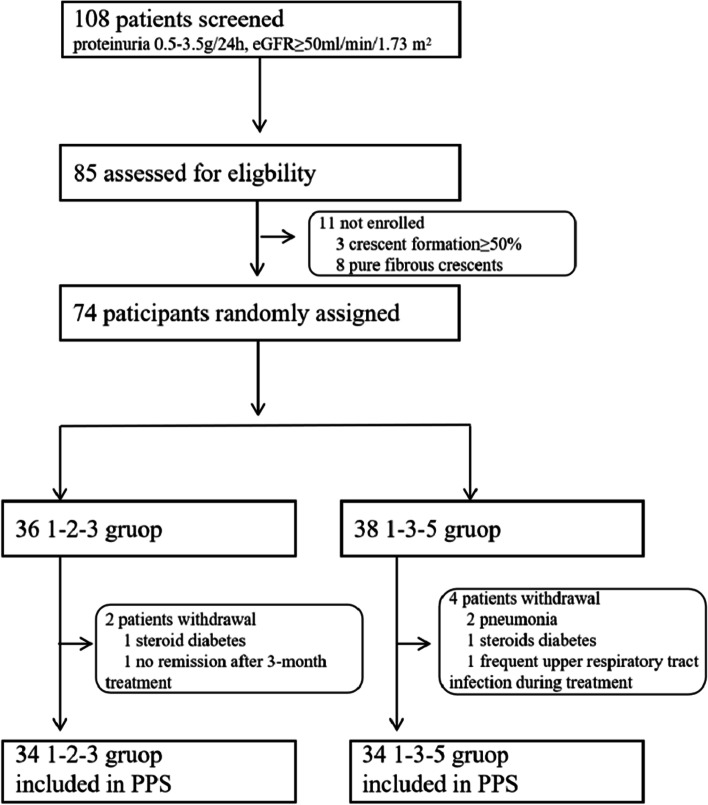
Enrollment flow diagram

There were initially 74 patients who participated in the pulse therapy session, including 36 cases in the 1–2-3 and 38 cases in the 1–3-5 group. However, 2 patients in the 1–2-3 voluntarily withdrew from therapy due to steroid diabetes and without remission after 3-month treatment, while 4 patients in the 1–3-5 groups withdrew due to pneumonia (*n* = 2), steroids diabetes (*n* = 1), and frequent upper respiratory tract infection during treatment (n = 1). (Fig. 1) Therefore, 68 patients (mean age = 31.74 ± 10.34 years; 33 males and 35 females) were included for analyses, 34 cases for each group. All 68 patients were followed up for 6 months. As shown in Table 1, there was no difference in proteinuria, serum albumin, serum creatinine, eGFR, MEST-C score, crescent proportion, and blood pressure between the 1–2-3 and 1–3-5 groups.

**Table 1:** Patient’s clinical characteristics

Variables	1–2-3 (*n* = 34)	1–3-5 (n = 34)	All (*n* = 68)	P
Age, years	29.65 ± 10.50	33.82 ± 9.90	31.74 ± 10.34	0.096
Sex, Male, n(%)	16 (47.06%)	17 (50.00%)	33 (48.53%)	0.808
Body mass index, Kg/m^2^	22.15 ± 8.51	22.78 ± 7.79	22.54 ± 8.12	0.896
Urine protein, g/24 h	2.04 ± 1.81	1.74 ± 0.93	1.89 ± 1.43	0.392
Serum albumin, g/L	36.34 ± 4.26	37.13 ± 7.24	36.62 ± 6.65	0.410
Serum creatinine, μmol/L	98.39 ± 38.20	105.51 ± 47.16	101.95 ± 42.74	0.496
eGFR, ml/min/1.73m^2^	89.86 ± 39.47	79.92 ± 31.23	84.89 ± 35.67	0.254
Lee grade, n(%)				0.127
II	3 (8.82%)	4 (11.76%)	7 (10.29%)	
III	19 (55.88%)	18 (54.55%)	37 (55.22%)	
IV	10 (29.41%)	9 (27.27%)	19 (28.36%)	
V	2 (5.88%)	3 (9.09%)	5 (7.46%)	
MEST-M, n(%)	34 (100.00%)	34 (100.00%)	68 (100.00%)	1.000
MEST-E, n(%)	9 (26.47%)	7 (20.59%)	16 (23.53%)	0.234
MEST-S, n(%)	11 (32.35%)	9 (26.47%)	20 (29.41%)	0.457
MEST-T, n(%)				0.563
T1	11 (32.35%)	9 (26.47%)	20 (29.41%)	
T2	3 (8.82%)	3 (8.82%)	3 (8.82%)	
MEST-C score, n(%)				1.000
C1	30 (88.24%)	30 (88.24%)	60 (88.24%)	
C2	4 (11.76%)	4 (11.76%)	8 (11.76%)	
Crescents(%)	14.66 ± 8.44	11.45 ± 9.37	13.05 ± 9.00	0.143
Follow-up period, months	6.00 ± 0.00	6.00 ± 0.00	6.00 ± 0.00	1.000
Blood pressure, mmHg				
Systolic at the start	126.75 ± 8.43	130.40 ± 9.25	129.58 ± 8.88	0.345
Diastolic at the start	72.60 ± 7.50	73.45 ± 8.21	73.10 ± 7.96	0.586
Systolic at the end	122.21 ± 7.61	124 ± 6.89	123 ± 7.21	0.611
Diastolic at the end	70.66 ± 6.89	72.26 ± 7.43	71.39 ± 7.01	0.802
RAAS inhibition, n(%)				
Prior to enrollment	18 (52.94%)	20 (58.82%)	38 (55.88%)	0.605
During follow-up	28 (82.35%)	29 (85.29%)	57 (83.82%)	0.780

*RAAS* renin-angiotensin system

### Changes in urine protein, serum creatinine, and eGFR

**Fig. 2:**
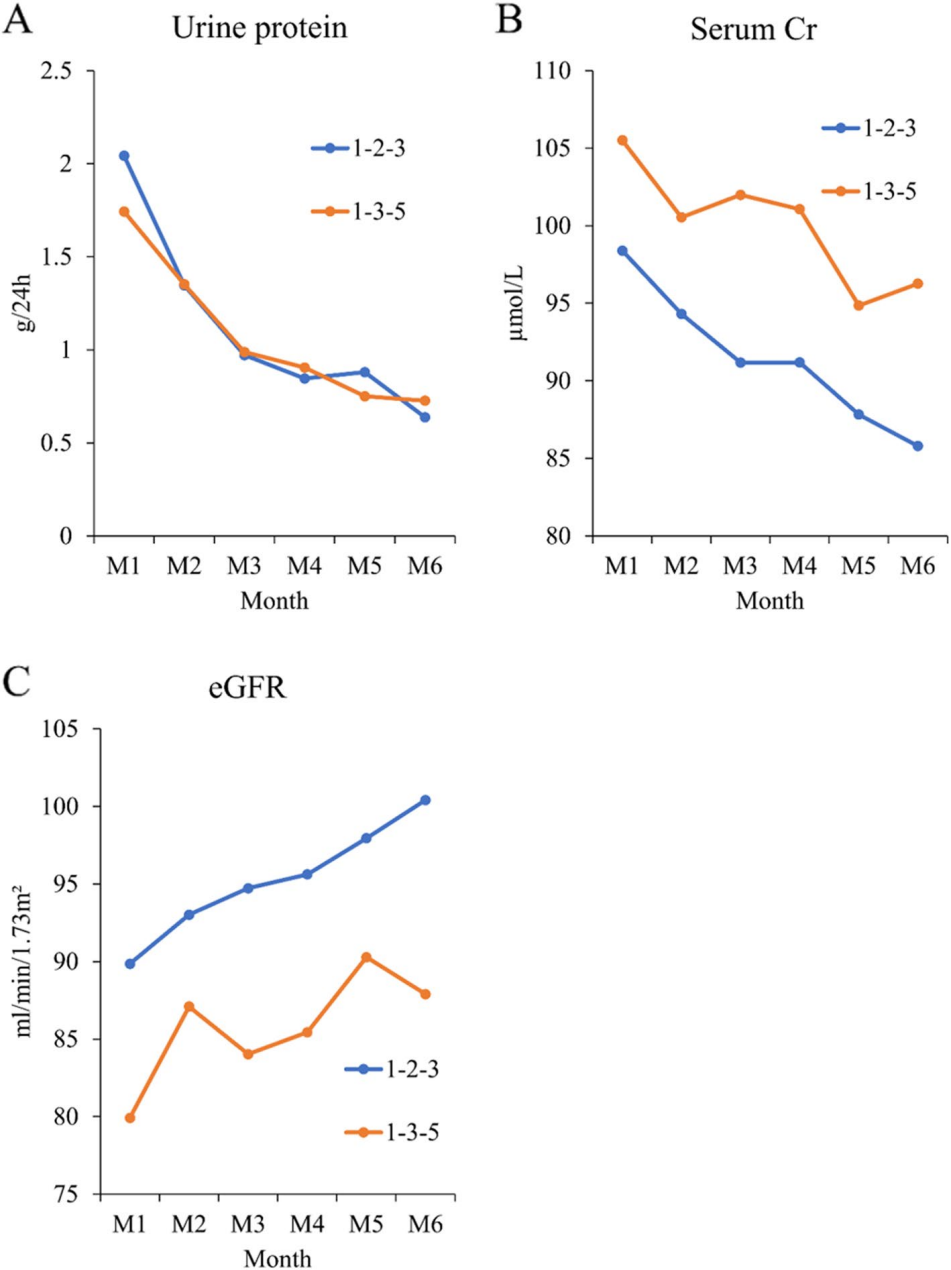
The changes of urine protein (**A**), serum creatinine (**B**), and eGFR (**C**) over time

The levels of urine protein, serum creatinine, and eGFR from month 1 to month 6 were compared between groups. As shown in Table 2 and Fig. 2, there was no significant difference in the three parameters at all time points between the 1–2-3 and 1–3-5 groups (all *P* > 0.05). However, the changes over time within-group or all patients were all significant (all *P* < 0.05, Table 2). As shown in Fig. 2, urine protein and serum creatinine had a decreasing trend, while eGFR had an increasing trend. Although there was no significance, the 1–2-3 group had descriptively lower creatinine and higher eGFR than the 1–3-5 group.

**Table 2:** Changes of urine protein, serum creatinine, and eGFR over time

Variables	1–2-3 (n = 34)	1–3-5 (*n* = 34)	All (*n* = 68)	P
Urine protein, g/24 h				
Month 1	2.04 ± 1.81	1.74 ± 0.93	1.89 ± 1.43	0.392
Month 2	1.35 ± 1.53	1.35 ± 0.88	1.35 ± 1.24	0.985
Month 3	0.97 ± 1.40	0.99 ± 0.83	0.98 ± 1.14	0.953
Month 4	0.85 ± 1.25	0.91 ± 0.79	0.88 ± 1.04	0.816
Month 5	0.88 ± 1.18	0.75 ± 0.74	0.82 ± 0.98	0.589
Month 6	0.64 ± 1.09	0.73 ± 0.79	0.68 ± 0.95	0.703
P among time-points	< 0.001	< 0.001	< 0.001	
Serum creatinine, μmol/L				
Month 1	98.39 ± 38.20	105.51 ± 47.16	101.95 ± 42.74	0.496
Month 2	94.31 ± 32.77	100.54 ± 40.69	97.43 ± 36.80	0.489
Month 3	91.18 ± 31.50	101.99 ± 39.44	96.59 ± 35.84	0.216
Month 4	91.19 ± 31.83	101.06 ± 39.48	96.13 ± 35.94	0.260
Month 5	87.83 ± 27.92	94.86 ± 34.09	91.34 ± 31.13	0.356
Month 6	85.79 ± 27.60	96.27 ± 32.00	91.03 ± 30.12	0.153
P among time-points	< 0.001	0.013	< 0.001	
eGFR, ml/min/1.73m^2^				
Month 1	89.86 ± 39.47	79.92 ± 31.23	84.89 ± 35.67	0.254
Month 2	93.02 ± 41.86	87.10 ± 46.13	90.06 ± 43.82	0.581
Month 3	94.73 ± 36.61	84.03 ± 40.92	89.38 ± 38.91	0.260
Month 4	95.62 ± 39.31	85.44 ± 41.45	90.53 ± 40.42	0.302
Month 5	97.95 ± 38.37	90.28 ± 38.94	94.12 ± 38.56	0.416
Month 6	100.41 ± 37.28	87.90 ± 44.56	94.16 ± 41.26	0.214
P among time-points	0.010	0.045	0.001

### Linear regression estimation of group factor under GEE models

The linear regression estimations of group factor to urine protein, serum creatinine, and eGFR including 6 time-points results under GEE models were performed. As shown in Table 3, there was no significant difference in urine protein, serum creatinine, and eGFR between the 1–2-3 and 1–3-5 groups (all *P* > 0.05). However, the estimated B value of serum creatinine (8.75) and eGFR (− 10.86) reflected the descriptive difference between groups as shown in Fig. 2.

**Table 3:** Linear regression estimation of group factor under GEE models

Group factor	Estimated B^a^	P
Urine protein, g/24 h		
1–2-3	ref.	–
1–3-5	−0.08 (−0.58 to 0.42)	0.752
Serum creatinine, μmol/L		
1–2-3	ref.	–
1–3-5	8.75 (−7.55 to 25.05)	0.293
eGFR, ml/min/1.73m^2^		
1–2-3	ref.	–
1–3-5	−10.86 (−27.93 to 6.22)	0.213

^a^B is the linear regression coefficient estimated in linear model

### Clinical outcomes and complications

**Fig. 3:**
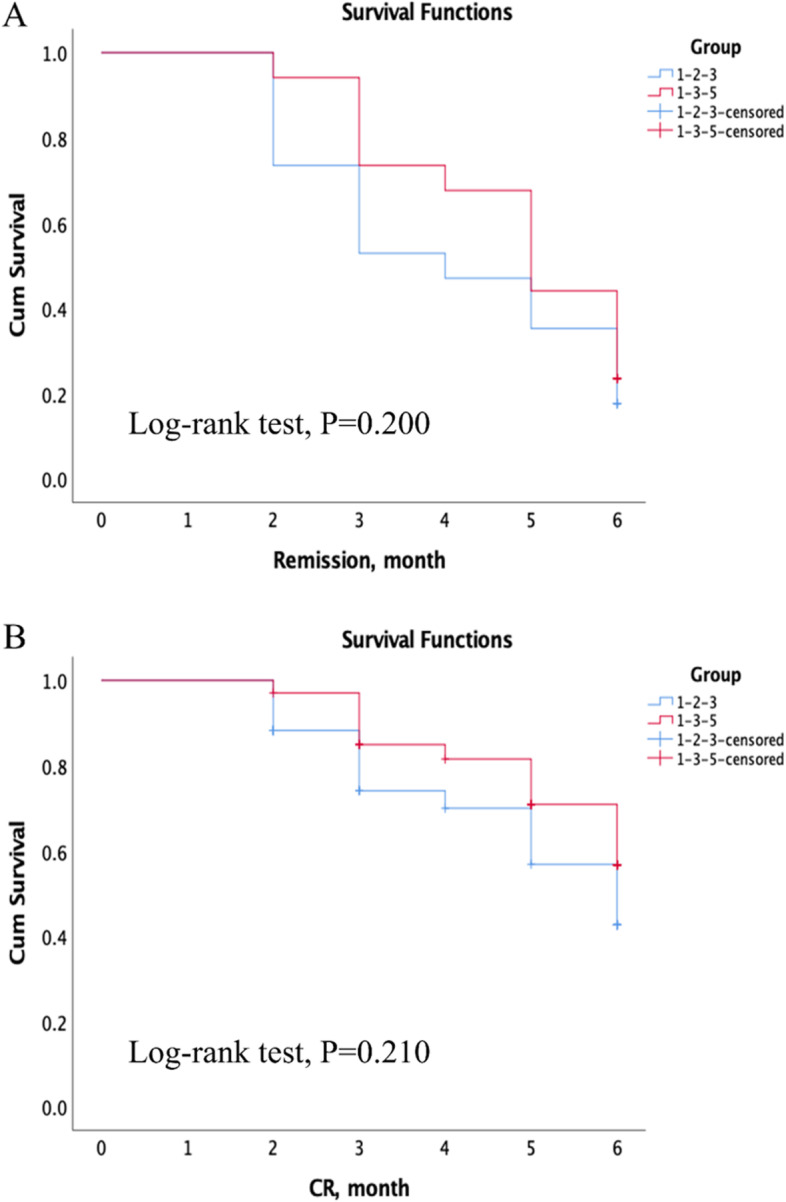
The Kaplan-Meier survival functions of 1–2-3 and 1–3-5 groups to total remission (CR + PR) (**A**), and CR only (**B**)

During the period of therapy, there were no patients that crossed-over to different arms. Table 4 shows the clinical outcomes after pulse therapy. Overall, there were 26 (38.24%) CR, 29 (42.65%) PR, and 13 (19.12%) NR of all patients. Remission (or total remission) was defined as CR + PR. The rates of remission in the 1–2-3 group and 1–3-5 group were 85.3 and 76.47%, respectively (*P* = 0.644). The mean period of remission was 4.04 ± 1.52 months, but that in the 1–2-3 group seemed shorter. No patient had serum creatinine increasing over 50% after 6-month therapy, and 2 patients had eGFR decreasing over 25% after therapy The Kaplan-Meier survival curve analysis also indicated that there was no difference in total remission (*P* = 0.200) and CR (*P* = 0.210) between the two groups (Fig. 3).

**Table 4:** Clinical outcomes

Variables	1–2-3 (n = 34)	1–3-5 (n = 34)	All (n = 68)	P
Remission status, n(%)				0.644
CR	14 (41.18%)	12 (35.29%)	26 (38.24%)	
PR	15 (44.12%)	14 (41.18%)	29 (42.65%)	
NR	5 (14.71%)	8 (23.53%)	13 (19.12%)	
Remission time, month	3.68 ± 1.59	4.42 ± 1.36	4.04 ± 1.52	0.071
Remission subtype, n(%)				
Total remission (CR + PR)	28 (82.35%)	26 (76.47%)	54 (79.41%)	0.549
CR (only)	15 (44.12%)	12 (35.29%)	27 (39.71%)	0.457
Renal function at month 6, n(%)				
Serum creatinine increasing over 50%	0	0	0	1.000
eGFR decreasing over 25%	1 (2.94%)	1 (2.94%)	2 (2.94%)	1.000

*CR* complete remission; *PR* partial remission; *NR* no response

Ten (13.51%) patients had different complications during the treatment period of all the patients. (Table 5) Six patients (8.11%) had encountered infections, including upper respiratory tract infection, pneumonia and herpes zoster. All the infections had happened during the second month during follow-up and been cured within 2 weeks. Four patients (5.41%) had got steroid diabetes which happened during the second or third month, and their doses of oral prednisone had been reduced to 0.25 mg/Kg/d on the third month of therapy. There was no significant difference in the rates of adverse events between groups.

**Table 5:** Clinical complications

Adverse events	PPS			SS		
	1–2-3(n = 34)	1–3-5(n = 34)	P1	1–2-3(*n* = 36)	1–3-5(*n* = 38)	P2
Steroid diabetes, n(%)	2 (5.88%)	0	0.493	3 (8.33%)	1 (2.63%)	0.351
Upper respiratory tract infection, n(%)	0	1 (2.94%)	1.000	0	2 (5.26%)	0.494
Pneumonia, n(%)	1 (2.94%)	0	1.000	1 (2.78%)	2 (5.26%)	1.000
Herpes zoster, n(%)	0	1 (2.94%)	1.000	0	1 (2.63%)	1.000
All, n(%)	3 (8.82%)	2 (5.88%)	1.000	4 (11.11%)	6 (15.79%)	0.737

P1 value for PPS, P2 value for SS

## Discussion

It has been suggested that the Pozzi protocol can significantly reduce proteinuria and protect against renal function deterioration in IgAN patients [10, 11]. However, the outcomes of glucocorticoid therapy for IgAN remain inconclusive and conflicting [17]. In recent years, large randomized controlled trials (RCTs) on corticosteroid therapy for IgAN patients, such as the STOP trial [18] and the TESTING trial [19], included patients with primary IgAN proteinuria (1.0–3.5 g/24 h) and glomerular filtration rate (eGFR) 20-120 ml/min/1.73m^2^ for corticosteroid therapy (0.6–1.0 mg /Kg/d), and the results show that the corticosteroid therapy is beneficial to the alleviation of proteinuria and the protection of the renal function of IgAN patients in the short term, but the server adverse effects of corticosteroid therapy are also noticed. It is recommended that corticosteroid therapy should be used with caution for IgAN patients. However, in these studies, patients with moderate to severely impaired renal function (20-30 ml/min/1.73m^2^) were included, and the influence of active pathological changes on the choice of treatment options and the prognosis was not considered.

Studies have suggested that corticosteroids and immunosuppressive therapy could be considered for IgAN patients with crescentic glomerulonephritis (kidney biopsy showed crescents in 50% of the glomeruli) [9, 20]. Nevertheless, for IgAN patients with crescents in lower than 50% of the glomeruli, there are no guidelines on the treatment options. According to the clinical database of our department (shown in Fig. 1), 78.70% of IgAN patients with proteinuria 0.5–3.5 g/24 h and eGFR ≥50 ml/min/1.73m^2^ combined with crescent formation, and 75.93% of which did not reach the diagnostic criteria of crescentic glomerulonephritis. Acute kidney injury may become chronic kidney disease after 3 months without intervention, we considered that more timely corticosteroid therapy, such as changing the timing of intravenous methylprednisolone in the Pozzi protocol from the 1st-3rd-5th months to the 1st-2nd-3rd, may have better therapeutic efficacy. Another retrospective study had demonstrated that corticosteroids protected the renal outcome and slowed the eGFR decline rate of IgAN patients with C1, it also decreased the eGFR decline rate even in those with initial proteinuria< 1 g/d [21].

In this study, we evaluated the effectiveness and safety of the treatment protocol of methylprednisolone intravenous infusion at the 1st-2nd-3rd months for the IgAN patients with crescents. The results showed that from month 1 to month 6, there was a decreasing trend in the levels of urine protein and serum creatinine and an increasing trend in eGFR in both groups. These results suggested that both the 1–2-3 and the 1–3-5 protocols can attenuate proteinuria and improve renal function in IgAN patients with crescents. Notably, the 1–2-3 group had lower creatinine and higher eGFR as compared with the 1–3-5 group throughout the 6-month follow-up, although the difference was not significant. The estimated B value of serum creatinine (8.75) and eGFR (− 10.86) also reflected the descriptive difference between groups. This result suggested compared to the Pozzi protocol (the 1–3-5 protocol), the 1–2-3 protocol seemed to have better effectiveness, especially for IgAN patients with crescents. However, the effectiveness remains to be further evaluated in a large trial.

Meanwhile, it’s important to pay attention to the appearance of adverse events of corticosteroids. In the literature, major side effects occurred in 6.2–35% IgAN patients enrolled in RCTs [18, 19, 22]. In our preliminary study results, 10 patients (13.51%) had encountered different complications during the treatment period. Six patients (8.11%) had encountered infections, including upper respiratory tract infection, pneumonia and herpes zoster and most of them were mild. All the infections had happened during the second month during follow-up four patients (5.41%) had got steroid diabetes which happened during the second or third month. There was no significant difference in the rates of other adverse events between groups.

In this study, the noteworthy adverse effect of glucocorticoid pulse therapy was steroid-induced diabetes. Studies have reported that the incidence of steroid diabetes varies from 1.5 to 55%. The shortest onset time of steroid diabetes is 7 days, and most steroid diabetes occurs 3–6 months after steroid therapy [23–28]. A study has shown that glucocorticoid therapy may produce undesired diabetogenic side effects through interactions with the regulation of glucose homeostasis which involved peripheral insulin resistance and impaired islet function, and the severity and progression of these alterations depend on several parameters, including dosage, period, and previous individual susceptibility [29]. In fact, the above-mentioned concept has been thought to be over-simplistic, and steroid diabetes is lack attention in previous studies on the glucocorticoid treatment of glomerulonephritis. More effort is necessary to minimize the disadvantages of glucocorticoid treatment, especially steroid-induced hyperglucagonaemia.

There are still some limitations to this study. As a single-center study, the sample size was relatively small. In addition, the follow-up duration was relatively short, only 6 months of the induction period. Nevertheless, our preliminary results still can provide a reference for the treatment options of IgAN patients with crescents. In the future, a multi-center study should be conducted to extend the observation time, from induction therapy to maintenance therapy, and to explore the effectiveness and safety of glucocorticoid therapy in IgAN with crescents.

## Conclusions

In summary, both the 1st-3rd-5th and 1st-2nd-3rd protocols can effectively alleviate proteinuria and protect renal function in IgAN patients with crescents. The 1st-2nd-3rd protocol seemed to have better effectiveness, although the differences were not significant.

## Data Availability

The datasets used and/or analysed during the current study available from the corresponding author on reasonable request.

## Abbreviations

IgAN: IgA nephropathy
ESRD: End-stage renal disease
eGFR: Estimated glomerular filtration rate
KDIGO: Kidney Disease Improving Global Outcome
CR: Complete remission
PR: Partial remission
NR: No response
PPS: Per protocol set
SS: Safety set
SD: Standard deviation
GEE: Generalized estimating equation
RCTs: Randomized controlled trials
